# Supplementary material for: Prevalence and inequality in persistent undiagnosed, untreated, and uncontrolled hypertension: Evidence from a cohort of older Mexicans
Source: PLOS Glob Public Health. 2021 Dec 16;1(12):e0000114. doi: 10.1371/journal.pgph.0000114 (PMC10021230; doi:10.1371/journal.pgph.0000114)
Supplement: S1 Table — (DOCX) [file pgph.0000114.s001.docx]

**S1 Table. Variables included in this principal component analysis for the wealth index.**

| **Assets & Income** | **Housing** |
| --- | --- |
| Television | Owned or rented dwelling |
| Security system | Type of floor dwelling |
| Cars | Type of wall dwelling |
| Electricity | Main source of drinking water |
| Bicycle | Type of toilet facility |
| Built-in kitchen sink | Type of fuel for cooking |
| Hot running water |  |
| Washing machine |  |
| Dish washer |  |
| Refrigerator |  |
| Housekeeper |  |
| Mobile |  |
| Bullock cart |  |
| Computer |  |
| HiFi or music centre |  |
| Livestock |  |
| Internet access at home |  |
| Motorbike |  |
| Second home |  |
| Own land or property |  |
| Own other valuable items |  |
| Regular income |  |
