# Supplementary material for: Prevalence and inequality in persistent undiagnosed, untreated, and uncontrolled hypertension: Evidence from a cohort of older Mexicans
Source: PLOS Glob Public Health. 2021 Dec 16;1(12):e0000114. doi: 10.1371/journal.pgph.0000114 (PMC10021230; doi:10.1371/journal.pgph.0000114)
Supplement: S2 Table — (DOCX) [file pgph.0000114.s002.docx]

**S2 Table. Characteristics of Wave 2 respondents aged 50 years and older years in 2014.**

|  | **Comparison sample –**  **observed in Wave 2** | **HTN Incomplete case sample – observed in Waves 1 & 2** | **Analysis (complete case) sample –**  **observed in Waves 1 & 2 and full item response on covariates** |
| --- | --- | --- | --- |
|  | **(n=2,998)** | **(N=1,254)** | **(n=945)** |
| **Continuous** | **Mean (SD)** | **Mean (SD)** | **Mean (SD)** |
| Age | 62.5 (9.3) | 71.1 (8.2) | 70.7 (8.0) |
| Systolic blood pressure | 138.8 (22.0) | 142.2 (23.9) | 141.8 (23.2) |
| Diastolic blood pressure | 78.9 (11.0) | 76.6 (11.3) | 76.6 (11.0) |
| **Categorical** | **No. (percentage)** | **No. (percentage)** | **No. (percentage)** |
| *Hypertension* |  |  |  |
| Yes | 1,675 (55.9) | 818 (65.2) | 609 (64.4) |
| No | 1,323 (44.1) | 436 (34.8) | 336 (35.6) |
| *Sex* |  |  |  |
| Female | 1,613 (53.8) | 764 (60.9) | 523 (55.3) |
| Male | 1,385 (46.2) | 490 (39.1) | 422 (44.7) |
| *Marital status* |  |  |  |
| Married / cohabiting | 2,105 (70.2) | 713 (56.9) | 625 (66.1) |
| Other | 893 (29.8) | 541 (43.1) | 320 (33.9) |
| *Location* |  |  |  |
| Urban | 2,356 (78.6) | 876 (69.9) | 645 (68.3) |
| Rural | 642 (21.4) | 378 (30.1) | 300 (31.8) |
| *Health insurance* |  |  |  |
| Yes | 2,508 (83.7) | 925 (89.2) | 845 (89.4) |
| No | 490 (16.3) | 112 (10.8) | 100 (10.6) |
| *Smoker status* |  |  |  |
| Smoker | 375 (12.5) | 123 (9.8) | 104 (11.0) |
| Non-smoker | 2,623 (87.5) | 1,131 (90.2) | 841 (89.0) |
| *Alcohol consumer* |  |  |  |
| Yes | 1,844 (61.5) | 547 (43.6) | 438 (46.3) |
| No | 1,154 (38.5) | 707 (56.4) | 507 (53.7) |
| *Weight* |  |  |  |
| Normal weight | 670 (23.2) | 336 (28.9) | 270 (28.6) |
| Overweight | 1,236 (41.2) | 482 (41.5) | 397 (42.0) |
| Obese | 1,065 (35.5) | 344 (29.6) | 278 (29.4) |

*Notes*. The analysis sample was observed in both waves and had full item response. The comparison sample was observed in Wave 2. Survey sampling weights were applied to the comparison sample to make it representative of the population aged 50 years and older in 2014.
