# Supplementary material for: Prevalence and inequality in persistent undiagnosed, untreated, and uncontrolled hypertension: Evidence from a cohort of older Mexicans
Source: PLOS Glob Public Health. 2021 Dec 16;1(12):e0000114. doi: 10.1371/journal.pgph.0000114 (PMC10021230; doi:10.1371/journal.pgph.0000114)
Supplement: S3 Table — (DOCX) [file pgph.0000114.s003.docx]

**S3 Table. Prevalence of hypertension and unaware, untreated, and uncontrolled hypertension, incomplete case sample.**

|  | **N=1,245** | | | | | | |  |
| --- | --- | --- | --- | --- | --- | --- | --- | --- |
|  | **Wave 1** | | **Wave 2** | | **Both waves** | | |  |
|  | **No.** | **(%)  [95% CI]** | **No.** | **(%)  [95% CI]** | | **No.** | **(%)  [95% CI]** | |
| All hypertension (HTN) | 797 | (63.6)  [60.1, 66.8] | 818 | (65.2)  [62.1, 68.2] | | 661 | (52.7)  [49.9, 55.5] | |
| HTN Undiagnosed | 388 | (30.9) [27.3, 33.0] | 479 | (22.0) [19.8, 24.3] | | 146 | (11.6) [9.9, 13.5] | |
| HTN Untreated | 458 | (36.5) [33.7, 39.5] | 336 | (26.8) [24.4, 29.3] | | 192 | (15.3) [13.4, 17.4] | |
| HTN Uncontrolled | 712 | (56.8) [53.4, 60.1] | 616 | (49.1) [45.9, 52.3] | | 459 | (36.6) [33.9, 39.3] | |

*Note.* As Table 3 in manuscript but using sample that does not exclude those with incomplete item response on covariates used in Table 6.
