# Supplementary material for: Prevalence and inequality in persistent undiagnosed, untreated, and uncontrolled hypertension: Evidence from a cohort of older Mexicans
Source: PLOS Glob Public Health. 2021 Dec 16;1(12):e0000114. doi: 10.1371/journal.pgph.0000114 (PMC10021230; doi:10.1371/journal.pgph.0000114)
Supplement: S4 Table — (DOCX) [file pgph.0000114.s004.docx]

**S4 Table. Transitions between hypertension states between Wave 1 and Wave 2, incomplete case sample.**

|  | **Wave 2, No. (row%)**  **[95% CI]** | | |
| --- | --- | --- | --- |
| **Wave 1** | No HTN | HTN Diagnosed | HTN Undiagnosed |
| No HTN | 300 (65.7)  [61.3, 69.8] | 63 (13.8)  [10.9, 17.3] | 94 (20.5)  [17.2, 24.4] |
| HTN Diagnosed | 32 (7.6)  [5.5, 10.5] | 352 (83.8)  [80.2, 86.9] | 36 (8.6)  [6.3, 11.5] |
| HTN Undiagnosed | 104 (27.6)  [22.7, 33.1] | 127 (33.7) [29.3, 38.3] | 146 (38.7)  [34.5, 43.1] |

|  | **Wave 2, No. (row %) [95% CI]** | | |
| --- | --- | --- | --- |
| **Wave 1** | No HTN | HTN Controlled | HTN Uncontrolled |
| No HTN | 300 (65.7)  [61.3, 69.8] | 36 (7.8)  [5.7, 10.8] | 121 (26.4)  [22.8, 30.6] |
| HTN Controlled | 10 (11.8)  [6.6, 20.2] | 39 (45.9)  [34.2, 58.0] | 36 (42.3)  [31.1, 54.5] |
| HTN Uncontrolled | 126 (17.7)  [14.7, 21.2] | 127 (17.8)  [15.1, 21.0] | 459 (64.5)  [60.1, 68.6] |

|  | **Wave 2, No. (row %)**  **[95% CI]** | | |
| --- | --- | --- | --- |
| **Wave 1** | No HTN | HTN Treated | HTN Untreated |
| No HTN | 300 (65.7)  [61.3, 69.8] | 55 (12.0)  [9.3, 15.5] | 102 (22.3)  [18.8, 26.2] |
| HTN Treated | 27 (7.9)  [5.6, 11.3] | 270 (79.6)  [75.5, 83.3] | 42 (12.5)  [9.6, 15.9] |
| HTN Untreated | 109 (23.8)  [19.7, 28.5] | 157 (34.3)  [30.1, 38.7] | 192 (41.9)  [37.6, 46.4] |

*Note.* As Table 4 in manuscript but using sample that does not exclude those with incomplete item response on covariates used in Table 6.
