# Supplementary material for: Prevalence and inequality in persistent undiagnosed, untreated, and uncontrolled hypertension: Evidence from a cohort of older Mexicans
Source: PLOS Glob Public Health. 2021 Dec 16;1(12):e0000114. doi: 10.1371/journal.pgph.0000114 (PMC10021230; doi:10.1371/journal.pgph.0000114)
Supplement: S5 Table — (DOCX) [file pgph.0000114.s005.docx]

**S5 Table. Transitions from hypertension diagnoses and treatment to control, respondents with hypertension in both waves.**

|  | **Wave 2, No. (row %)**  **[95% CI]** | |
| --- | --- | --- |
| **Wave 1** | HTN Controlled | HTN Uncontrolled |
|  | (n=166) | (n=495) |
| HTN Diagnosed | 127 (32.7)  [28.2, 37.5] | 261 (67.3)  [62.4, 71.2] |
| HTN Undiagnosed | 39 (14.3)  [10.6, 18.9] | 234 (85.7)  [81.0, 89.3] |
| HTN Treated | 107 (34.3)  [29.2, 39.7] | 205 (65.7)  [60.3, 70.8] |
| HTN Untreated | 59 (16.9)  [13.3, 21.2] | 290 (83.1)  [78.8, 86.7] |

*Note.* Using sample that does exclude those with incomplete item response on covariates used in Table 6.

|  | **Wave 2, No. (row %)** | |
| --- | --- | --- |
|  | **[95% CI]** | |
| **Wave 1** | HTN Controlled | HTN Uncontrolled |
|  | (n=125) | (n=358) |
| HTN Diagnosed | 97 (34.4)  [29.0, 40.1] | 185 (65.6)  [59.9, 70.9] |
| HTN Undiagnosed | 28 (13.9)  [9.8, 19.5] | 173 (86.1)  [80.5, 90.2] |
| HTN Treated | 87 (37.5)  [31.5, 43.9] | 145 (62.5)  [56.0, 68.5] |
| HTN Untreated | 38 (15.1)  [11.2, 20.1] | 213 (84.9)  [79.9, 88.8] |

*Note.* Using sample that does exclude those with incomplete item response on covariates used in Table 6.
