# Supplementary material for: Prevalence and inequality in persistent undiagnosed, untreated, and uncontrolled hypertension: Evidence from a cohort of older Mexicans
Source: PLOS Glob Public Health. 2021 Dec 16;1(12):e0000114. doi: 10.1371/journal.pgph.0000114 (PMC10021230; doi:10.1371/journal.pgph.0000114)
Supplement: S6 Table — (DOCX) [file pgph.0000114.s006.docx]

**S6 Table. Averaged marginal effects (ME) on probabilities of transitioning to diagnosed, treated, and controlled hypertension.**

|  | **ME [95% CI] (P-value)** | | |
| --- | --- | --- | --- |
|  | **Undiagnosed to diagnosed** | **Untreated**  **to treated** | **Uncontrolled to controlled** |
|  | **(n=286)** | **(n=340)** | **(n=526)** |
| **Sex** |  |  |  |
| Female | *Ref* | *Ref* | *Ref* |
| Male | -0.18 [-0.33, -0.04]  (0.014) | -0.16 [-0.28, -0.03] (0.015) | -0.09 [-0.17, -0.01] (0.049) |
| **Age (years)** | 0.01 [-0.00, 0.01] (0.129) | 0.00 [-0.00, 0.00] (0.461) | 0.00 [-0.00, 0.01]  (0.578) |
| **Cohabiting** |  |  |  |
| Yes | *Ref* | *Ref* | *Ref* |
| No | -0.13 [-0.27, 0.02]  (0.085) | -0.07 [-0.19, 0.06] (0.288) | -0.05 [-0.13, 0.04] (0.313) |
| **Living area** |  |  |  |
| Urban | *Ref* | *Ref* | *Ref* |
| Rural | 0.01 [-0.12, 0.14] (0.832) | 0.02 [-0.10, 0.06] (0.764) | -0.03 [-0.12, 0.05] (0.409) |
| **Health insurance** |  |  |  |
| Health insurance | *Ref* | *Ref* | *Ref* |
| No health insurance | -0.15 [-0.35, 0.05] (0.129) | -0.02 [-0.18, 0.15] (0.827) | -0.14 [-0.27, 0.00]  (0.059) |
| **Wealth status** |  |  |  |
| Richest 50% | *Ref* | *Ref* | *Ref* |
| Poorest 50% | 0.02 [-0.10-0.15] (0.727) | 0.06 [-0.05-0.18] (0.263) | 0.05 [-0.02, 0.13] (0.172) |
| **Body weight** |  |  |  |
| Normal weight | *Ref* | *Ref* | *Ref* |
| Overweight | 0.10 [-0.05, 0.25] (0.200) | -0.03[ -0.17, 0.10] (0.624) | -0.03 [-0.13, 0.07] (0.602) |
| Obese | 0.15 [-0.03, 0.32] (0.093) | 0.04 [-0.11, 0.19] (0.635) | 0.08 [-0.02, 0.18] (0.102) |
| **Smoker** |  |  |  |
| No | *Ref* | *Ref* | *Ref* |
| Yes | 0.27 [0.09-0.46] (0.003) | 0.15 [-0.02, 0.32] (0.074) | 0.04 [-0.08, 0.16] (0.509) |
| **Alcohol consumption** |  |  |  |
| No | *Ref* | *Ref* | *Ref* |
| Yes | -0.01 [-0.16, 0.13] (0.872) | 0.01 [-0.13-0.14] (0.932) | 0.05 [-0.04, 0.14] (0.282) |

*Notes*. Probit estimates of marginal effects averaged over the respective samples. Samples include only those with undiagnosed, untreated, or uncontrolled hypertension in Wave 1, respectively. Dependent variable is 1 if participant moves to diagnosed, treated, or controlled in Wave 2, respectively. Sample is split into two wealth groups, rather than three as in Table 6, because of smaller sample sizes.
