# Supplementary material for: Prevalence and inequality in persistent undiagnosed, untreated, and uncontrolled hypertension: Evidence from a cohort of older Mexicans
Source: PLOS Glob Public Health. 2021 Dec 16;1(12):e0000114. doi: 10.1371/journal.pgph.0000114 (PMC10021230; doi:10.1371/journal.pgph.0000114)
Supplement: Attachment — Submitted filename: Reponse to Reviewers.docx [file pgph.0000114.s007.docx]

We thank both reviewers and the editor for their knowledgeable and relevant comments, which have helped considerably to strengthen our study, particularly the presentation of results. Please find below our point-by-point responses. The page numbers refer to the “clean” document.

**Additional Editor Comments (if provided):**

**0.1 There is a conceptual problem in determining the hypertensive individuals in the study. Hypertension is a chronic condition that may not be cured but can be controlled. Therefore, an individual, once (e.g., wave-1) diagnosed as hypertensive can either have controlled or uncontrolled hypertension; but cannot be categorized as not having hypertension at a later period (e.g., wave-2). Hence, the outcome variable, “HTN in both waves”, is technically incorrect. All hypertensive individuals in wave-1, should be categorized as hypertensive in wave-2 as well. Some of them may have hypertension under control in wave-2 either by taking anti-hypertensive medication or adopting healthy lifestyle (e.g., physical exercise, food habit, etc.) or both. Please address this issue and revise the analysis as required.**

Response. While clinically diagnosed hypertension is, indeed, a chronic condition that can be controlled but not cured, we do not observe this outcome. We use blood pressure (BP) measured on a single occasion in the participant’s home at the time of the survey interview, plus the participant’s self-report of ever having been diagnosed with high BP, to classify each respondent as having hypertension or not. This is consistent with the approach taken in many (cross-sectional) studies of awareness, treatment, and control of hypertension. Since hypertension would usually be diagnosed by a medical professional on the basis of BP measurements on more than one occasion, the approach will result in classification errors. Some participants with BP ≥140/90 mm Hg at the time of the survey will be false positives. Recognizing this, it would be inappropriate to classify a participant as having hypertension in Wave 2 only because they gave a BP reading above the thresholds on a single occasion at the time of Wave 1. A strength of our longitudinal study is that we can partially take account of classification errors by focusing on those with BP measurements above the thresholds in both waves, and on those with BP above thresholds in Wave 1 and self-reported diagnosis in Wave 2. These are much less likely to be false positives. Hence, we should be more concerned about those who were classified as having hypertension in both waves and yet remained undiagnosed, untreated, or uncontrolled.

Note that if a participant had BP ≥140/90 mm Hg in Wave 1 and had BP below the thresholds in Wave 2 (possibly due to medication or lifestyle changes that achieved BP control) we would classify that participant as having hypertension in both waves if they reported ever having been diagnosed with high BP in Wave 2.

To avoid giving the impression that participants who had been clinically diagnosed with hypertension at the time of Wave 1 could possibly no longer have the condition in Wave 2, we no longer refer to participants as “hypertensive”. Instead, we always preface with “classified as”, as in “classified as having hypertension (HTN)”. To clarify and justify our approach, we have added the following in the Method section.

*“**While we recognized that clinically diagnosed hypertension is a chronic condition, we did not classify a participant as necessarily having hypertension in Wave 2 if they were classified with the condition in Wave 1. The reason was that we did not observe clinical diagnoses made on the basis of BP measurements on multiple occasions. Measured BP ≥140/90 mm Hg on a single occasion could be a false positive. By classifying participants in each wave using only their measured BP and self-reported diagnoses from that wave, we avoided contaminating Wave 2 classifications with Wave 1 measurement errors.” - pages 7-8*

We have also added the following in the results section:

*“This percentage is lower than the prevalence in either wave because some respondents (n=107) transitioned from being classified as hypertensive in Wave 1 to normotensive in Wave 2 (see Table 4). These transitions arise for two reasons. First, measured BP on a single occasion, in a non-clinical setting, can be above the hypertension thresholds in Wave 1 and below the thresholds in Wave 2. If such respondents report in Wave 2 that they have never been diagnosed with high BP/hypertension, then they will not be classified as having hypertension in Wave 2. These cases may have been false positives in Wave 1. Second, a participant could report having ever been diagnosed with hypertension in Wave 1 but in Wave 2 report never having had such a diagnosis. Such reporting implies a measurement error, either in Wave 1 or Wave 2.” - page 12*

The Discussion section acknowledges the limitation that we (in common with all hypertension awareness, treatment, and control studies) do not observe clinical diagnosis based on BP measurements on multiple occasions. It also argues that a strength of our longitudinal perspective is the ability to partially take account of measurement errors by focusing on those consistently classified as having hypertension.

**0.2 I personally think the advantage of having a longitudinal data was under-utilized in achieving the aim of the study (i.e., identifying the socioeconomic characteristics associates with hypertension care cascade - diagnosed, treated, controlled). For example, the analyses do not offer what share of undiagnosed hypertensive individuals in wave-1 were diagnosed in wave-2 and how that differ by the respondent's sociodemographic characteristics. Similarly, those were diagnosed but not treated in wave-1, how their sociodemographic characteristics were associated with receiving treatment in wave-2. As pointed out by Reviewer 1, please emphasis on what the longitudinal analyses revealed on this issue. It appears that the longitudinal data were treated as repeated cross-sectional data. Please clarify and justify.**

Response. Figure 2 and Table 4 show the share of undiagnosed participants in Wave 1 who were diagnosed in Wave 2 (32.9%). More generally, the transition analyses reported in this figure and table exploit the longitudinal dimension of the data. We did not treat the data as merely repeated cross-sections. The right-hand column (Both waves) of both Table 3 and 5, as well all columns of Table 6 rely on the longitudinal nature of the data.

We are grateful for the suggestion to obtain further insights from the longitudinal structure of the data by examining how the probabilities of moving from undiagnosed to diagnosed, untreated to treated, and uncontrolled to controlled varied with socioeconomic and other characteristics. We have explained these additional analyses in the Method section:

*“We also conducted a probit regression to estimate how the probability of transitioning from undiagnosed hypertension in Wave 1 to diagnosed in Wave 2 varied with covariates. The sample used for this analysis consisted of those undiagnosed in Wave 1. We conducted analogous analyses to examine variation in the transition probabilities between untreated and treated and between uncontrolled and controlled.” - page 9*

The results from these analyses are presented in Supplementary File Table S6 and summarized in the

Result section:

*“Analyses of variation in the probabilities of transitioning from undiagnosed to diagnosed, untreated to treated, uncontrolled to controlled revealed that males were significantly less likely to make each of these transitions (Supplementary file S6). Smokers were significantly more likely to move from undiagnosed to diagnosed.” - Page 17*

To save on space, we have placed this table of results in the Supplementary Material. Should you believe that it warrants inclusion in the main paper, we would be happy to make that change.

We have edited the introduction and discussion to place more emphasis on the longitudinal nature of the analyses and what this reveals*.*

**0.3 Please clarify whether the complex survey weights were used in the analysis, if not then provide justification.**

Response. We did not use sampling weights in our main analyses because the weights supplied were intended to make each cross-sectional sample representative of the population in the respective year. We examine a cohort of participants observed in both waves. There were no weights available to make this cohort representative of a defined population. Sampling weights were used to make the cross-sectional comparison sample described in Table 2 representative of the population aged 50+ in 2014. This allowed us to assess how the sociodemographic composition of the cohort analyzed compared with that population. There were some differences that should be taken into account when interpreting our results. We now mention this in the Method section.

*“We did not apply sampling weights since these were not available at cohort level. We assessed representativeness of the cohort by comparing its sociodemographic composition with that of the full Wave 2 cross-sectional sample with sampling weights representative of the population aged 50 years and older in 2014.” - Page 19*

The results of the comparison referred to are presented in Table 2. We have now elaborated on the implications of the comparison in the Discussion.

*“Comparison with the Wave 2 cross-section sample weighted to be representative of the population aged 50 years and older showed that the cohort was older, and, consequently, had higher rates of hypertension, rural dwellers, and health insurance coverage, and it was less likely to be cohabiting and to drink alcohol. Our results should be interpreted with these differences in mind. They do not necessarily hold for the population of Mexico aged 50 years and older in 2014, although they are likely to be more representative for an older population. Selective attrition could also potentially leave the cohort unrepresentative with respect to unobserved characteristics that are related to hypertension and its management.” - Pages 22-23*

**0.4 Provide a discussion on how the findings of this paper are comparable with hypertension care in other countries.**

Response. We have rewritten the entire Discussion section to better connect the findings to evidence from other countries. For example:

*“In the study cohort, hypertension was slightly more prevalent among the wealthier. This adds to already conflicting evidence from Latin America regarding socioeconomic inequality since it is reported that individuals with a lower SES had a higher risk for an elevated blood pressure, while another study summarizes recent evidence from LMIC settings with the majority of the studies confirming the positive relationship between socioeconomic status and chronic conditions (including hypertension) (41,42). Furthermore, evidence from a low-income setting in Mexico revealed that using two different aspects of SES showed an inverse association with elevated blood pressure (43). In our sample, we found that less wealthy individuals were slightly more likely to have persistent undiagnosed hypertension and more likely to have persistent untreated and uncontrolled hypertension, however, these differences were not significant. Previous evidence showed that the performance of health systems in LMICs regarding the management of hypertension was poor: not even halve of those with hypertension were diagnosed, only one third were taking medication and 10% had their blood pressure under control (44).” - Pages 21-22*

**Reviewer 1**

**This paper uses data from Waves 1 and 2 of the Mexican sample of the WHO SAGE to estimate the prevalence of persistent forms of hypertension, the transitions between different care states, and differences across socioeconomic groups. I think this perspective is extremely important, especially, as the authors point out, because the current literature in this area is dominated by cross-sectional analyses that may lead to misleading policy conclusions. I think the findings are important and have provided my comments on what I believe needs to be addressed below.**

**In the spirit of transparency, my colleagues are currently working on similar analyses based on data from four countries (one of which is Mexico). This perceived "competition" however, has not influenced my comments and I am keen to see this paper published as well! If it is useful to the authors, their paper is here:**

**https://www.medrxiv.org/content/10.1101/2021.04.30.21256391v1**

**Some of their findings on Mexico differ from the ones presented here and I think it would be interesting to understand why.**

Response. Thank you for generously sharing your colleagues’ work. We have cited it in the Discussion, highlighted differences from our findings and offered some possible explanations for these differences.

1. **Introduction**

**Major comments**

1.1 **The motivations for the study and how they link to the analyses the authors present is a bit unclear to me. While I understand the link between the prevalence of long-term uncontrolled HTN analyses and the motivation that risks rise with duration spent uncontrolled, I wasn't sure from reading the intro what the purpose of the transition analyses was.**

Response. We agree and have rewritten the Introduction to make the motivation more consistent with the analyses we undertook. In particular, we now list three limitations of cross-sectional analyses of hypertension awareness, treatment, and control, along with corresponding advantages of the longitudinal analyses we conducted. Specifically concerning the purposes of the transition analyses, we have added the following in the Introduction.

*“Second, cross-sectional analyses cannot determine the rates at which people move from undiagnosed to diagnosed, untreated to treated, and uncontrolled to controlled, and how these transition probabilities vary with sociodemographic characteristics. A cross-sectional approach also does not reveal reverse transitions from treated to untreated hypertension and controlled to uncontrolled, both of which indicate failures in hypertension management and treatment adherence.” – page 4*

*“This can help in targeting interventions on those likely to be exposed to the greatest health risk arising from living with persistent uncontrolled hypertension. <….> A longitudinal perspective makes it possible to measure and compare rates of transitions between hypertension states that can be used to identify points of weakness in hypertension management and the characteristics of those most likely to face the gaps.” – page 4*

1.2 **Related to point 1, it would be useful to directly tie how policy insights regarding hypertension improvements might be different when examining longitudinal, rather than cross-sectional, evidence. The authors might provide one or two examples of scenarios where cross-sectional and longitudinal results lead to differing conclusions.**

Response. We have added a couple of such scenarios in the Introduction.

*“If the probabilities of becoming diagnosed, treated, and controlled differ across sociodemographic groups, then the persistently undiagnosed, untreated, and uncontrolled will differ from those identified in these states with a snapshot, cross-sectional study. Longitudinal and cross-sectional analyses would then lead to targeting of improvements in hypertension screening and management on different groups. Longitudinal and cross-sectional estimates of undiagnosed, untreated, and uncontrolled hypertension may also point to different priorities with respect to the design of such improvements**. For example, a cross-sectional analysis may find a high rate of undiagnosed hypertension. But if a longitudinal analysis revealed less persistence in this state than in the state of uncontrolled hypertension, then it may be better to prioritize treatment and its adherence over screening.” – page 4*

**Minor comments**

1.3 **First page, second paragraph: I would specify which ages the 31% prevalence is estimated for (among 30+, 50+, etc).**

Response. We have added that this percentage refers to the population aged 18 years and older.

1.4 **Same paragraph: The estimates on the current state of HTN control in Mexico was based on data from 2010. If possible, providing these estimates based on more recent data would be more informative.**

Response. We have now given estimates of HTN prevalence and undiagnosed, untreated, and uncontrol from 2016, and stated that year explicitly.

1.5 **Final paragraph: In the statement on what the study aims are, the authors just mention the prevalence of long-term undiagnosed analyses and not the transition analyses. Since that is an important part of the paper, my suggestion would be to mention that as well.**

Response. We agree and have included this.

*“**This study aimed to estimate the prevalence and socioeconomic distribution of hypertension that remained undiagnosed, untreated, and uncontrolled for at least five years among Mexicans aged 50 years and older and to estimate rates of transition from those states to diagnosis, treatment, and control.”- page 4*

2. **Methods**

**Major comments**

2.1 **Do the authors have any insights from the published reports of the SAGE data on why the response rates were so low/which groups were more likely to be missed? This would help understand which population these results are most relevant for.**

Response. We have added the below information on the response rate. Unfortunately, no further information on response rates and characteristics of those missing was provided in the SAGE reports.

*“**Wave 1 had a relatively low response rate of 53%. Response was lower for middle-aged adults aged 50-59 years (42%) than for younger adults aged 18-49 years (58%). The low response rate has been attributed to the short time available for field work, which left little time to revisit sampled households that did answer during the initial visit in this wave (6), but no further information on the average characteristics of those missing has been made available. An interval of six-seven years between Wave 0 and Wave 1 also contributed to a high rate of attrition and a low response in the latter wave (6).” - page 6*

2.2 **The authors should justify why their sample was those who were aged 50+ in wave 2 rather than wave 1. Why not start with those who were age 50+ in wave 1? To me that seems more logical and may lead to a larger final sample.**

Response. We have clarified this by adding the below to the manuscript. Selecting those aged 50+ in Wave 2 gives a larger (cohort) sample than would be achieved by selecting those aged 50+ in Wave 1.

*“The SAGE sample was designed, after weighting, to be nationally representative for the population aged 50 years and older at the time of each wave. We restricted the analysis sample to this age range. To conduct longitudinal analyses, we had to restrict the sample to respondents observed in both Wave 1 and Wave 2. To maximize the size of this cohort, we selected respondents aged 50 years and older in Wave 2 who also participated in Wave 1. Some of these respondents were therefore slightly younger than 50 in Wave 1.” - Page 6*

Note that if we had selected those aged 50+ in Wave 1 and followed them forward, we would have excluded those aged <50 in Wave 1 and would not have gained any additional participants relative to the sample selection we used (age 50+ in Wave 2). The approach taken produced the largest cohort subject to the restriction that the cohort had be to aged 50+ in either Wave 1 or Wave 2.

2.3 **I think there may be some inconsistencies in the definition of hypertension. The authors state it is based on measured BP or reported diagnosis and to have taken medication. What happens to those who have a measured BP in the controlled range, that report diagnosis, but do not report treatment? To me, these individuals should still be considered hypertensive but that doesn't seem to be the case based on the current definition.**

Response. Thank you for drawing our attention to a mistake in our stated definition of hypertension, which was inconsistent with the definition we had actually used in our calculations. Apologies for that. We have rewritten the definition as follows.

*“Blood pressure (BP) was measured using a Boso Medistar Wrist Blood Pressure Monitor Model S during a home visit (6). Three measurements were taken, with a minimum of one minute between each. Each participant was asked: “Have you ever been diagnosed with high blood pressure (hypertension)?” A positive response was followed with: “Have you been taking any medications or other treatment for it during a) the last 2 weeks, b) the last 12 months?” We classified a participant as having hypertension (HTN) if a) the last two measurements gave a mean systolic BP ≥140 mm Hg or mean diastolic BP ≥ 90 mm Hg, or b) they reported ever having been diagnosed with high BP (13,26).”- Page 7*

2.4 **Related to this, it is not clear from the text that the undiagnosed, untreated, and uncontrolled rates are among those with hypertension and not overall. I mentioned this below, but I think the authors may want to present results for this sub forms of hypertension non-conditional on being hypertensive, since that is more aligned with the goal of describing the prevalence of long-term uncontrolled or untreated HTN that the authors put forth in the intro.**

Response. Thank you for this suggestion. We agree and have now used one analysis sample throughout the paper and conducted all analyses unconditional on hypertension status. This indeed better aligns with our study objective.

**Minor comments**

2.5 **I found the description of the sampling procedures, inclusion and exclusion, and other sample restrictions to be a bit hard to follow. I would suggest that the authors tighten this section up. I was missing, for example, information on what the stages and strata of the sampling strategy were and how many units (households, districts, etc.) were targeted at each stage of sampling.**

Response. We have rewritten the description of the sampling to improve clarity and added information on the nature of the strata, the sampling stages and the number of units targeted at each stage.

*“**The sample for Wave 1 (November 2009 - January 2010) was based on the 2003 WHO World Health Survey (WHS) for Mexico (hereafter, Wave 0). A total of 96 strata were defined over 32 states and three levels of urbanicity (rural, urban, and metropolitan) (6). A nationally representative sample was obtained in Wave 0 by conducting cluster random sampling with Basic Geo-Statistical Areas forming the primary sample units (PSUs). In total, 40,000 households were randomly sampled (24). To obtain the Wave 1 sample, probability sampling was used to select 211 PSUs from the 797 sampled in Wave 0 (25). In each selected rural and urban PSU, all Wave 0 individuals who had been aged 50 years or older in 2003 were included in the Wave 1 target sample (ibid). In each of the selected metropolitan PSUs, a random sample of 90% of individuals aged 50 years and older in 2003 were included. In addition, a systematic sample of 1000 individuals from Wave 0 who had been aged 18-49 across all selected PSUs were included as the primary sample.*

*Wave 1 had a relatively low response rate of 53%. Response was lower for middle-aged adults aged 50-59 years (42%) than for younger adults aged 18-49 years (58%). The low response rate has been attributed to the short time available for field work, which left little time to revisit sampled households that did answer during the initial visit in this wave (6), but no further information on the average characteristics of those missing has been made available. An interval of six-seven years between Wave 0 and Wave 1 also contributed to a high rate of attrition and a low response in the latter wave (6).*

*SAGE Wave 2 was conducted in July-October 2014. The target sample included all individuals who participated in Wave 1, plus those aged 50 years and older in 2014 who were not in the Wave 0 (or Wave 1) sample but who lived in a household that included someone from that sample (6). The Wave 2 response rate was 83% for households and 81% for individuals.” - page 5-6*

2.6 **The phrasing of "hypertension, undiagnosed, untreated, and uncontrolled hypertension" is somewhat awkward throughout the paper. It may be clearer to call the current "hypertension" variable "All hypertension" such that the analysis would correspond to "all hypertension and by whether they had undiagnosed hypertension, untreated hypertension, or uncontrolled hypertension."**

Response. We agree and have changed this in the tables and, according to another suggestion, we describe hypertension in the text as “classified with hypertension”.

3. **Results**

The figures and tables are quite nice, good job by the authors on presenting the information.

**Major comments**

3.1 **The trends overtime in hypertension are presented without confidence intervals or other measures of uncertainty. For example, the authors state that 62% of the cohort was hypertensive in wave 1 and 64% in wave 2. Based on the size of the confidence intervals, however, I don't think the authors can interpret this as a detectable or real increase.**

Response. We agree and no longer refer to a rise in hypertension prevalence between Wave 1 and Wave 2, and we have now given 95% CI in parentheses after each point estimate that is mentioned in the text. We have also added 95% CIs to Table 4.

3.2 **Why is the prevalence of hypertension in both waves lower than the prevalence of hypertension in either wave? I think this would occur if many people with hypertension in wave 1 became normotensive in wave 2, but this should be specified.**

Response. Indeed, some participants transition from being classified as with hypertension in Wave 1 to not being classified as such in Wave 2 (n=107). These transitions arise for two reasons. First, measured BP can be above the hypertension thresholds in Wave 1 and below the thresholds in Wave 2. If such participants report in Wave 2 that they have never been diagnosed with high BP/hypertension, then they will not be classified as having hypertension in Wave 2. As we argue in response to the Editor’s comment 0.1, this seems a sensible approach given that we do not observe clinically diagnosed hypertension in Wave 1. BP measurements are taken on a single occasion in each wave, and some who have measured BP above the thresholds at the time of the survey will be false positives for hypertension. Our approach does not imply that anyone with clinically diagnosed hypertension can be cured of the condition. The second reason a participant could transition out of the hypertension classification would be because they report ever having been diagnosed with the condition at Wave 1 but at Wave 2 they report never having had such a diagnosis. Such reporting implies a measurement error. We do not know whether the error occurs in Wave 1 or Wave 2. An advantage of having longitudinal data is that we can identify inconsistent reporting, which gives an idea of the extent to which cross-sectional analyses are ridden with measurement error. For transparency, we believe it is best to classify on the basis of the data measured and reported in each wave and to record all the resulting transitions. We have clarified our approach in the Methods section as follows.

*“While we recognized that clinically diagnosed hypertension is a chronic condition, we did not classify a participant as necessarily having hypertension in Wave 2 if they were classified with the condition in Wave 1. The reason was that we did not observe clinical diagnoses made on the basis of BP measurements on multiple occasions. Measured BP ≥140/90 mm Hg on a single occasion could be a false positive. By classifying participants in each wave using only their measured BP and self-reported diagnoses from that wave, we avoided contaminating Wave 2 classifications with Wave 1 measurement errors.” - pages 7-8*

In the Results section, we have added:

*“**This percentage is lower than the prevalence in either wave because some respondents (n=107) transitioned from being classified as hypertensive in Wave 1 to normotensive in Wave 2 (see Table 4). These transitions arise for two reasons. First, measured BP on a single occasion, in a non-clinical setting, can be above the hypertension thresholds in Wave 1 and below the thresholds in Wave 2. If such respondents report in Wave 2 that they have never been diagnosed with high BP/hypertension, then they will not be classified as having hypertension in Wave 2. These cases may have been false positives in Wave 1. Second, a participant could report having ever been diagnosed with hypertension in Wave 1 but in Wave 2 report never having had such a diagnosis. Such reporting implies a measurement error, either in Wave 1 or Wave 2.” - page 12*

3.3 **The description of the treatment and control results is challenging to interpret because the types of hypertension are conditional on being hypertensive in that wave. To me, the goal the authors put forth in the intro of estimating the prevalence of persistent forms of undiagnosed hypertension would imply non-conditional estimates. For example, the authors might present:**
a. **What was the all-hypertension prevalence in w1, in w2, and how many people had hypertension in both waves?**
b. **What was the undiagnosed hypertension prevalence (non-conditional, e.g. those with undiagnosed hypertension among the entire sample) in w1, in w2, and the prevalence of undiagnosed hypertension in both waves?**
c. **Same for untreated and uncontrolled (e.g. those with untreated hypertension among the entire sample and those with uncontrolled hypertension among the entire sample).**

**This type of presentation would be easier to interpret and align more naturally with the motivation of the paper. It would also be more consistent with the transition results presented in the next section, since those show transitions, for example, from no HTN to treated HTN, implying a w1 sample that also includes normotensives.**

Response. Thank you for this suggestion, which we have implemented by reporting now unconditional estimates throughout the paper (instead of conditional estimates). Your specific a, b, and c suggestions can be found now in Table 3, on page 13.

3.4 **The authors present results on transitions in the text that have to be calculated based on the results in the table. For example, the authors state that "More than three fifths of the remainder became hypertensive without being diagnosed." For this, I had to first add the HTN diagnosed and HTN undiagnosed in w2 sample sizes then divide the 78 people in the HTN undiagnosed in w2 category by this total. I would suggest that the authors first present the numbers in the table, before presenting more aggregated results. For example, the authors may say "Among those that not hypertension in wave 1, 22% became hypertensive but undiagnosed and 13.5% hypertension and diagnosed by wave 2. This means that nearly 3/5ths (22% / 22%+13.5%) of those that became hypertensive were undiagnosed. I found the figure very easy to interpret but the corresponding text challenging to follow.**

Response. We have rewritten the text interpreting Figure 2 and Table 4 on pages 13-14 as suggested.

3.5 **The authors should present confidence intervals or some other measure of uncertainty around the transition estimates - many of them are based on very small samples and right now showing the numbers alone gives a false sense of precision and definitiveness to the results.**

Response. We have added the 95% CI for the transition estimates (Table 4).

3.6 **A similar comment to 3 but for the multivariate results. I think this set of analyses would be far easier to interpret if it was done among the entire sample rather than subsetted to just the those with hypertension in both waves.**

Response. We agree and now present the multivariable results based on the full sample, Table 5, pp 17.

**Minor comments**

3.7 **Page 9, top of the page: The authors say "Within the cohort, there were improvements in hypertension diagnosis, treatment and control by Wave 2" without providing the corresponding numbers. These should be included to give the reader a sense of by how much things have improved.**

Response. We have included this and now state the following.

*“We estimated that in Wave 1, 30.3% (95% CI, 27.2 to 33.5) of the cohort had undiagnosed hypertension, 36.0% (95% CI, 32.8 to 39.3) had untreated hypertension, and 55.7% (95% CI, 52.1 to 59.2) had uncontrolled hypertension.* *In Wave 2, the prevalence rates of undiagnosed, untreated, and uncontrolled hypertension were estimated to be 22.2% (95% CI, 19.7 to 24.9), 27.1% (95% CI, 24.4 to 30.0), and 48.7% (95% CI, 45.4 to 52.0) respectively. Between the two waves, there was a significant reduction in the prevalence of hypertension that was undiagnosed (P=0.000), untreated (P=0.000), and uncontrolled (P=0.000).” – page 12*

3.8 **Why dichotomize the poverty index for the multivariate analyses? I think the authors are throwing out a lot of variation by cutting it at 50%.**

Response. Earlier the small sample size constrained the number of groups that could be compared. However, the larger sample that now results from using estimated unconditional on being hypertensive has allowed us to use wealth terciles instead of the dichotomized variable*.* The updated results can be found in Table 6, page 17.

3.9 **What is the justification for including the not-cohabiting variable as a predictor? I think a short section where the authors justify each predictor and how knowing how htn varies across this predictor could inform targeting would be useful in the methods section. Otherwise, these variables appear to be somewhat cherry picked.**

Response. Our hypothesis is that those living alone may be less likely to become aware of their hypertension, and they may have lower treatment adherence, resulting in lower BP control. To save on space, we have not referred to this specific entire hypothesis. We briefly justify all covariates included in the analysis as potentially related to either hypertension risk or to hypertension care access.

*“****Covariates***
*We examined associations between persistent undiagnosed, untreated, and uncontrolled hypertension and both sociodemographic and lifestyle characteristics that may plausibly have been related to the risk of hypertension or with access to health services that deliver hypertension care. Specifically, we examined associations with sex and age that are risk factors for hypertension and with cohabiting status, rural/urban location, wealth (index), and health insurance that may each be related with access to care (28,29). In addition, we examined associations with smoking, alcohol consumption, and Body Mass Index (BMI) that may each be related to hypertension risks (28,30,31). BMI was calculated from height and weight measured by a healthcare professional at the time of the interview. We categorized respondents as: normal weight (BMI <25.0), overweight (25.0-29.9) and obese (>29.9) (32). Very few respondents had a BMI lower than 20.0 (n=19), we included them in the “normal weight” category.” – page 8*

4. **Discussion**
   4.2 **There are two points in the discussion that should be expanded and brought out more:**
   a. **What do the longitudinal results reveal that the cross-sectional don't, and how does this difference matter for policy decision making?**
   b. **What might explain some of the patterns that the authors see (especially the differences by sex, rural/urban, etc)? Bringing in literature from other studies here**

**As of now, the discussion has a lot of re-hashing of the results rather than a discussion of why the authors see what they see and what the implications are.**

Response. We have completely rewritten the Discussion to address both points as well as the repetition of results, which are well taken. On the two points specifically, we provide below some examples of what we have incorporated.

*“Our longitudinal analysis revealed that well over one third of those classified as having undiagnosed hypertension were still in this state five years later, more than two fifths of those initially untreated remained untreated, and over three fifths of those initially with uncontrolled hypertension failed to achieve BP control by the end of the period…….” – page 19*

*“Our approach allowed for reclassifications from hypertensive to normotensive between waves and we found that such transitions are far from uncommon. Approximately, these are as common as moving to a diagnosed, treated, or controlled state.” – page 20*

*“The transition rates from undiagnosed to diagnosed, and untreated to treated are like in our study close to 30%, while we find a tree times higher rate for treatment continuity. In line with our work, they find men and rural dwellers to be less likely to advance forward through the continuum of hypertension care. The difference in treatment continuity might be driven by differences in the average characteristics of both cohorts. The cohort used by Mauer et al. (34) is derived from the Mexican Family Life Survey which also includes those aged 40 to 49 years old and had it first wave a few years earlier (2005). The second wave was apparently collected over a prolonged period of time (2009 till 2012). Those timing differences might have resulted in a lower observed treatment continuity given that the cohort was followed over a longer and less strictly defined time period.” – page 19-20*

4.3 **In the limitations, the authors state that there were differences between the sample and the Mexican population due to non-response, missing data, and loss-to-follow-up. It would help the reader if the authors could go one step further and characterize the population that the results are most relevant to.**

Response. We compared our analysis sample (cohort) to the full Wave 2 cross-section sample that is weighted to be representative of the populated aged 50+ in Table 2. We have now stated this more specific in the Discussion section and summarized the characteristics by which the samples differ.

*“Comparison with the Wave 2 cross-section sample weighted to be representative of the population aged 50+ revealed that the cohort was older, and, consequently, had higher rates of hypertension, rural dwellers, and health insurance coverage, and it was less likely to be cohabiting and to drink alcohol. Our results should be interpreted with these differences in mind. They do not necessarily hold for population of Mexico aged 50 years and older in 2014, although they are likely to be more representative for an older population.” – pages 22-23*

**Reviewer 2**

The study aims to estimate prevalence and socioeconomic inequalities in persistent undiagnosed, untreated, and uncontrolled hypertension using waves 1 (2009/10) and 2 (2014) of the WHO Study on Global AGEing and adult health (SAGE) that followed a cohort of Mexicans aged 50+ years.

Strengths: Longitudinal dataset

My comments are below

1. **Introduction**
   1.1 **Not sure what is the context of the reference of undiagnosed hypertension discussed in the first paragraph in the introduction. Is it in the context of developing countries or developed countries?**

Response. We agree and have clarified this point as follows.

*“Worldwide, there are substantial gaps in diagnosis, treatment, and control of hypertension (2–7) signaling failures to prevent CVDs and avert millions of premature deaths (8).” - page 3*

1.2 **Introduction needs further motivation regarding the role of socioeconomic inequalities in undiagnosed, untreated, and uncontrolled hypertension in the population of interest. It seems that role of socioeconomic inequity is key here but no attention has been paid in the introduction to motivate why SES inequity could contribute to undiagnosed, untreated, and uncontrolled hypertension in this population.**

Response. We have added a paragraph as proposed.

*“In high-income countries, hypertension tends to be more prevalent among lower socioeconomic groups (14). In low- and middle-income countries (LMIC), evidence on the socioeconomic gradient in hypertension is mixed, which may reflect changes in the gradient as countries move through the epidemiological transition (2,13,15,16). There is evidence, however, that the socially disadvantaged in LMIC have worse access to hypertension care (5,17) and so potentially suffer great ill-health as a consequence of uncontrolled hypertension. More effective and equitable targeting of hypertension screening and treatment requires improved understanding of the sociodemographic groups that face the greatest barriers in accessing these services.” - page 3*

**2. Method**

**2.1 What is the final sample size that includes both wave 1 and Wave 2 respondents?**

Response. The analysis sample size is 945. Previously, we used two samples for different parts of the analysis. One included all those observed in Wave 1 and Wave 2 and had full item response on covariates. The other was a subset of respondents that had hypertension. Following a suggestion from Reviewer #1, we now use the first sample throughout and indicate this in the Sample description of the Results section as shown below. We show the selection of this sample in Figure 1, and also indicate sample size in all relevant tables.

*“**In this cohort, valid BP measures were obtained in both waves for 1,254 (72%), and 945 (54%) had full item response on all measures and variables used in the analyses. We present results obtained from the latter, analysis sample. Estimates of prevalence and transition rates obtained from the larger sample with BP measures in both waves (BP sample) were highly consistent and are given in the Supplementary files (Tables S2 – S4).” – page 10*

**2.2 Wave 1 response rate seems to be really low (51%); did author perform any test that nonresponse rate in Wave 1 could contribute to bias in the final sample? In other words, are there any differences in individual characteristics between those who responded in wave 1 and those who did not?**

Response. We agree that the response rate is relatively low. In Table 2, we compared the characteristics of the cohort used for the analyses with those of the full Wave 2 cross-section that is weighted to be representative of the 2014 population aged 50+ (N=1,254). We describe the differences in the Sample section of the Results, and added these differences in the Limitations section of the Discussion. Please also see the responses to comments 2.1 and 4.3 of Reviewer #1.

**3. Results**

**3.1 From Table 2, it seems that there are some differences in prevalence of hypertension and other individual characteristics (location, drinking alcohol etc.) between analytic sample that combined both wave and 2 and comparison sample wave 2 only. Why is that? Authors need to offer implications of these differences and how that could contribute to the results?**

Response. The Sample section of the Results, and now also the Discussion, explains that the differences are likely to mainly arise because the analysis sample is eight years older. The average age in the analysis sample is higher because the sample includes only those who participated in both waves. In Wave 2, new participants were added to the sample, and they were younger. The weighted Wave 2 cross-section is representative of the population aged 50+ in 2014. Our cohort, as with all cohorts, it not representative of a population of a given age (range) alive at a point in time. This is a consequence of longitudinal analysis. We have now emphasized the differences between the samples and issued due warning about the inference that can be made from our findings in the Discussion.

**“**Comparison with the Wave 2 cross-section sample weighted to be representative of the population aged 50 years and older showed that the cohort was older, and, consequently, had higher rates of hypertension, rural dwellers, and health insurance coverage, and it was less likely to be cohabiting and to drink alcohol. Our results should be interpreted with these differences in mind. They do not necessarily hold for the population of Mexico aged 50 years and older in 2014, although they are likely to be more representative for an older population.**”** *- pages 22-23*

3.2 **Is there any statistically significant difference between prevalence of hypertension in wave 1 and wave 2?**

Response. We added p-values from tests of equality of prevalence rates between waves in the text. Prevalence of all hypertension does not differ significantly. The prevalence of each of undiagnosed, untreated, and uncontrolled hypertension does differ significantly.

3.3 **Table 5 needs some additional clarification—not sure if I follow the relationship between socioeconomic inequality and hypertension prevalence as author discussed on page 12.**

Response. We agree and have rewritten the interpretation of Table 5.

*“**Table 5 shows concentration indices that measure wealth-related inequality in each hypertension indicator. The positive concentration indices imply that a hypertension classification was more prevalent among wealthier respondents in each wave and that wealthier respondents were more likely to be classified as hypertensive in both waves. However, all of the 95% confidence intervals include zero so there was no evidence of statistically significant inequality in hypertension prevalence. The next three rows of the table show concentration indices for undiagnosed, untreated, and uncontrolled hypertension in each wave and in both waves. All the point estimates of these concentration indices are negative, indicating that poorer respondents were more likely to have undiagnosed, untreated, and uncontrolled hypertension. However, all of the 95% confidence intervals include zero, and thus the inequality apparent in the sample was not statistically significant.” #page 16*

3.4 **What is the outcome variable of the multivariate analysis (hypertension diagnosed in both waves)? Authors need to offer clear definition of the outcome measure before discussing multivariate analysis. Also, what was rationale of having this outcome that having hypertension in both waves? In general, if some has hypertension, it will likely be the case that after 5 years that person will most likely to have hypertension as well. It will be rather interesting to see, if someone is undiagnosed or not diagnosed with hypertension in wave 1, what are the likelihood that the person will be diagnosed with hypertension in wave 2 or remain undiagnosed (although had it) in wave 2?**

Response. We have added a description of the outcome variables used in the multivariate analyses. The three outcomes of the multivariable analyses are: persistent (from Wave 1 to Wave 2) undiagnosed, untreated, and uncontrolled hypertension. In the first submission, these outcomes were defined conditional on having hypertension, and we also estimated a model for having hypertension in both waves. We agree that the latter model is not too interesting and so we dropped it from Table 6. Following a suggestion from Reviewer #1, the other three outcomes – persistent undiagnosed, untreated, and uncontrolled hypertension are now unconditional, i.e. 1 if in the state in both waves and 0 otherwise.

The rationale for these outcomes is that we presume that there is concern about people living with hypertension that is undiagnosed, untreated, or uncontrolled for an extended period (five years in our study). These are bad outcomes that can be expected to have negative health consequences. There is likely to be interest in identifying which type of individuals are most likely to experience these outcomes in order that they can be targeted by hypertension care teams.

Thank you for the suggestion to look at variation in probabilities of transitioning from undiagnosed to diagnosed, untreated to treated, and uncontrolled to controlled. We have implemented this, included the results in Supplement Table S6 and mention the results in the manuscript.

We have described both analyses and defined the respective outcomes more precisely in the Methods section as follows.

*“**We estimated probit models of persistent (from Wave 1 to Wave 2) undiagnosed, untreated, and uncontrolled hypertension and used them to obtain averaged marginal effects that indicated by how much the probability of remaining in each of these states for at least 5 years varied with covariates. We also conducted a probit regression to estimate how the probability of transitioning from undiagnosed hypertension in Wave 1 to diagnosed in Wave 2 varied with covariates. The sample used for this analysis consisted of those undiagnosed in Wave 1. We conducted analogous analyses to examine variation in the transition probabilities between untreated and treated and between uncontrolled and controlled.” - page 9*

3.5 **What is “pp?” I assume it means “percentage point”? Again for the other outcome variables also some combination between wave would be interesting to examine. Also, please provide p-values in addition to CI for the probit regression results and discuss statistical significance of the results.**

Response. We have now defined pp as percentage point upon first use (page 17) and have added p-values to Table 6 and discuss statistical significance of marginal effects. Please also see our response to your comment 3.4 regarding the helpful suggestion to study transitions between both waves.

3.6 **It was not clear why only a handful of control variables were measured in the analysis, I believe the survey includes a host of control variables. Need description of these variables as well. For example, marital status, education etc. why not controlled in the analysis?**

Response. The sample is somewhat small and becomes smaller when more covariates are added due to incomplete item response. This is the reason, for example, that we did not control for education. We did control for marital status (cohabiting). We have now justified all covariates included in the analysis as potentially related to either hypertension risk or to hypertension care access.

*“****Covariates***
*We examined associations between persistent undiagnosed, untreated, and uncontrolled hypertension and both sociodemographic and lifestyle characteristics that may plausibly have been related to the risk of hypertension or with access to health services that deliver hypertension care. Specifically, we examined associations with sex and age that are risk factors for hypertension and with cohabiting status, rural/urban location, wealth (index), and health insurance that may each be related with access to care (28,29). In addition, we examined associations with smoking, alcohol consumption, and Body Mass Index (BMI) that may each be related to hypertension risks (28,30,31). BMI was calculated from height and weight measured by a healthcare professional at the time of the interview. We categorized respondents as: normal weight (BMI <25.0), overweight (25.0-29.9) and obese (>29.9) (32). Very few respondents had a BMI lower than 20.0 (n=19), we included them in the “normal weight” category.” – page 8*

4. **Discussion**
   **4.1 currently discuss section seem to be the repetition of the results section, I would suggest author to discuss in the context of current literature and implications for clinical decision-making and health outcomes.**

Response. We agree and have completely rewritten the Discussion to address this point and a similar comment made by Reviewer #1. We have removed repetition of results, focused on the added value of our longitudinal analysis relative to cross-sectional studies of hypertension diagnosis, treatment, and control, and better placed our work in the context of current literature. We have also increased the discussion of policy implications arising from the longitudinal approach.
