# Supplementary material for: Prevalence and inequality in persistent undiagnosed, untreated, and uncontrolled hypertension: Evidence from a cohort of older Mexicans
Source: PLOS Glob Public Health. 2021 Dec 16;1(12):e0000114. doi: 10.1371/journal.pgph.0000114 (PMC10021230; doi:10.1371/journal.pgph.0000114)
Supplement: Attachment — Submitted filename: Response to Reviewers.docx [file pgph.0000114.s008.docx]

**Response to Editor’s comments.**

Comments are in italics.

*Thank you for addressing the concerns raised by the reviewers. However, it appears that the longitudinal analysis, which is core to this paper, requires further improvement. The introduction was heavily built on the necessity of the longitudinal analysis to better understand the status of the hypertension management and assessing the needs accordingly. The analyses, however, did not adequately exploit the longitudinal techniques. For example, figure 2 and tables 3 and 4 basically provide descriptive statistics observed over time.*

**Response**. We use longitudinal data to estimate prevalence of persistent undiagnosed, untreated, and uncontrolled hypertension, and to identify correlates of these persistent states. In doing this, we would suggest that the paper makes a useful contribution. It moves a very large literature beyond point-in-time estimates of undiagnosed, untreated, and uncontrolled hypertension. We choose techniques that can deliver the paper’s objectives. If they do so, we would suggest that they are appropriate. The figure and tables referred to do indeed present descriptive statistics. In fact, we view the entire paper, including the regressions, as an exercise in descriptive analysis. This is entirely consistent with all the literature on hypertension awareness, treatment, and control to which we contribute. Please see the final response for the reason that descriptive analysis is sufficient in this context.

*A proper longitudinal analysis would require estimating panel models to report how odds of being diagnosed, treated, or having hypertension under control changes over time. A simple way of doing that is to estimate a panel probit model with a year dummy (e.g., d=1 if wave 2 and 0 if wave 1). The coefficient (or avg. marginal effect) of the year dummy would capture the change across waves. An advanced model could be panel multinomial logit with mutually exclusive outcomes such as undiagnosed, diagnosed but not treated, treated but not controlled, and controlled.*

**Response**. With respect, we would suggest that a “proper analysis” is one that delivers credible estimates of the parameters of interest. We are interested (for reasons given in the Introduction) in estimating prevalence of persistent hypertension states and their correlates. We believe that the transition matrix analysis, along with concentration indices and multivariable regression (probit) models of the persistent hypertension states do provide credible estimates of these parameters. Panel models would serve a different purpose. For example, as suggested, such a model could be used to estimate the change over time in the prevalence of a hypertension state conditional on covariates. (Please note that the unconditional change in prevalence can be observed from Table 3.) But this is not a parameter that interests us. We want to know the prevalence of persistent undiagnosed (/untreated/uncontrolled) hypertension, not the change in the prevalence of undiagnosed hypertension. Note that no change in the latter prevalence could be consistent with two very different scenarios. In one, there is 100% persistence: all who were undiagnosed remain undiagnosed, and nobody moves into this state. In the other, there is 0% persistence: all who were undiagnosed leave this state, and the same fraction of people move into this state. As we argue in the paper, there are good (health) reasons to be interested in the extent to which there is persistence in undiagnosed, untreated, and uncontrolled hypertension. Given, this motivation, we use techniques that deliver the relevant parameters.

For the same reasons, a panel multinomial logit would not estimate the parameters of interest. Further, we presume (from what follows) that the suggestion is to use a conditional/fixed effects logit model. To estimate that, we would have to drop from the sample precisely those observations that we are interested in: those who do not change state between waves. This is enough to convince us that this would not be a good idea: we are interested in persistence. But consider further that with this approach marginal effects would not be identified because the individual fixed effects are not identified.

*Unfortunately, as mentioned previously, without any proper longitudinal empirical analysis, the longitudinal perspective story is not viable. Similar is the case for the longitudinal analysis with respect to sociodemographic characteristics. The panel structure of the data is merely utilized when a probit model is estimated rather than a panel model that would exploit the within person change over time. Table 6 is a cross-section and repeated cross-section analysis and does not provide any longitudinal perspective. Table S6 is not a proper longitudinal analysis as it does not apply required empirical techniques to offer insights on within or between changes. Please revise the analysis utilizing proper longitudinal techniques.*

**Response**. Please note that it would not be possible to estimate relationships between a (point-in-time) hypertension state and socio-demographics (if one wanted to do that) by exploiting only within person changes because many of the socio-demographics we study are time invariant, or have relatively little time variation. In any case, this is not what we want to do. We are interested in socio-demographics that are correlated within remaining in a hypertension state. We are interested in these correlations because they can be useful for targeting groups that are most vulnerable to gaps in hypertension care. For this targeting purpose, it is not necessary to estimate causal effects. In targeting, a characteristic can serve as a marker (or tag) for another (unobserved) characteristic that has a causal effect. We do not interpret the regression estimates as causal. This is consistent with the whole literature on hypertension awareness, treatment, and control that seeks to identify who to target.

We appreciate that our use of the term “longitudinal analysis” may have given rise to expectations that are inconsistent with the paper’s objectives. To many, the term simply refers to analysis of longitudinal data. This is the way in which we were using it. But we recognize that for some others it may invoke specific analysis that allows for time invariant individual-specific effects -- panel data models, as they are called in some disciplines. To avoid any such confusion, we have dropped the term entirely from the paper. We now refer to our use (analysis) of longitudinal data, as opposed to that of cross-sectional data. In places, we claim to take a “longitudinal perspective”. In addition, we have checked that the motivation and statement of the objectives of the paper in the introduction are consistent with what the paper delivers.

We do very much appreciate these suggestions for changes to the paper, not least because they gave us the opportunity to further clarify what it delivers. In many contexts, the suggested analysis would be useful. But it would not be useful in realizing the objectives of this paper, and it would lose the paper’s focus on persistent undiagnosed, untreated, and uncontrolled hypertension, which is what distinguishes it from the literature.
