# Supplementary material for: Prevalence and inequality in persistent undiagnosed, untreated, and uncontrolled hypertension: Evidence from a cohort of older Mexicans
Source: PLOS Glob Public Health. 2021 Dec 16;1(12):e0000114. doi: 10.1371/journal.pgph.0000114 (PMC10021230; doi:10.1371/journal.pgph.0000114)
Supplement: Attachment — Submitted filename: Response to Reviewers.docx [file pgph.0000114.s009.docx]

**Response to editor’s comments**

**Author’s response:**

We thank the editor for the opportunity to further revise our manuscript in response to the thoughtful and useful feedback on the last version. The comments have helped us to better align the motivation set out in the introduction with the study’s stated aims and findings.

This note explains the revisions made in response to each comment. Comments are in italics. Our responses are in bold. Quotes from the paper are in bold italics. Page numbers refer to the “clean” version of the manuscript.

*I thank the authors to clarify their position on the analyses and scopes of the paper. There were indeed some different expectations based on the part of introduction that states limitations of using cross-sectional data and benefits of using longitudinal data in the introduction section as follows:

The introduction states, “First, it does not inform of how long hypertension remains undiagnosed, untreated, and uncontrolled.” – answering this question warrants survival type analysis, which was not an analysis offered in this paper, nor would that been feasible given the nature of data. What this paper offers are how many of the undiagnosed patients remained undiagnosed after five years, or how many of the untreated remained untreated after five years, which is very different from how long hypertension remained undiagnosed or untreated.*

**Response. We have revised the text to read as follows.**

**“*First, it does not provide information about persistent undiagnosed, untreated, and uncontrolled hypertension. Given that the risks of severe health consequences rise steeply with the duration of exposure to uncontrolled hypertension (22), it is important to establish prevalence of the condition that remains undiagnosed, untreated, and uncontrolled for an extended period of time and how these prevalence rates vary with sociodemographic characteristics*.” p. 3-4**

*The introduction states, “Second, with cross-sectional data one cannot determine the rates at which people move from undiagnosed to diagnosed, untreated to treated, and uncontrolled to controlled, and how these transition probabilities vary with sociodemographic characteristics.” – the standard expectation in this regard, particularly from the policy perspective, is to know how the odds of transition were associated with certain sociodemographic characteristics. While the paper provides simple transition rates, it does not offer how the actual transition rates differs across sociodemographic groups. The multivariate analysis presented in table 6 does not tell us how x% transition rate from wave-1 to wave-2 varies across wealth status or across urban and rural areas.*

**Response. We have changed the statement of the first limitation to include lack of evidence on how prevalence rates of persistent of hypertension states vary with sociodemographics (see previous response). Further, we rewrote the second limitation to refer only to evidence on transition rates, as follows.**

**“*Second, a cross-sectional approach does not allow estimation of rates of transition from undiagnosed to diagnosed, untreated to treated, and uncontrolled to controlled hypertension, nor can it reveal reverse transitions from treated to untreated and controlled to uncontrolled, both of which indicate failures in hypertension management and treatment adherence*.” p.4**

*The introduction states, “Third, analyses of cross-sectional data are particularly prone to finding false positive cases of hypertension as a result of relying on blood pressure measurement on a single occasion. These false positives will bias cross-section estimates of undiagnosed, untreated, and uncontrolled hypertension upwards.” – this paper clearly does not solve the problem of false positive outcomes as hypertension were not clinically diagnosed and there are overlapping across the two waves in determination of hypertensive status.*

**Response. We have deleted these two sentences. The point was that with longitudinal data one can identify survey respondents who are identified as having undiagnosed (/untreated/uncontrolled) hypertension in one wave and who have blood pressure below the hypertension thresholds and are not diagnosed or treated in a subsequent wave. These are likely to have been false positives in the first wave. The sample proportion of these cases provides an indication of the prevalence of false positives. However, we realize that this explanation is too convoluted for the introduction, and without it a reader may get the impression that we identify false positives by comparing survey measurement of blood pressure with clinical diagnosis. For that reason, we decided to drop the point entirely.**

*Then it states, “All three of these limitations can be addressed with longitudinal data.” – building the expectation that the analyses presented in this paper will address all these shortcomings of a typical cross-sectional analysis of hypertension care cascade. The analyses, however, did not adequately encompass any of the advantages mentioned about longitudinal data in the introduction.*

**Response. After making the revisions explained above, we believe that we now correctly claim to address the two limitations of analyses based on cross-sectional data we identify. We have changed the text to read as follows.**

**“*These limitations can be addressed by following a cohort over time to identify the proportion and type of people who remain undiagnosed, untreated, and uncontrolled for an extended period, as well as rates of transition to more favorable states*.” p.4**

*After a long presentation on the benefits of using longitudinal data, the aim of the study was stated as “to estimate the prevalence and socioeconomic distribution of hypertension that remained undiagnosed, untreated, and uncontrolled for at least five years among Mexicans aged 50 years and older and to estimate rates of transition from those states to diagnosis, treatment, and control.” – which didn’t offer significant improvement to any of the “three limiting aspects”.*

**Response. After revising the claimed limitations of analyses based on cross-section data, we believe that the stated aims of the study do address these limitations. That is, we aimed to fill gaps in evidence from cross-section analyses by estimating prevalence of persistent undiagnosed, untreated, and uncontrolled hypertension, how these prevalence rates vary with sociodemographic characteristics, and rates of transition to diagnosed, treated, and controlled hypertension. We identify that this evidence is missing and then state the aim of the study to provide it. The statement also makes the aim specific to the older population of Mexico.**  *I appreciate your explanations and do agree with your views regarding the selective scope of the study aims and their execution. However, I feel certain segments of the paper are not aligned with this aim and requires revision. In the context of the stated aim of this paper, I will be happy recommending this article for publication given the following concerns are addressed:*

**Response. We thank you for pushing us to better align the study’s motivation with what it delivers. With the revisions made, the study’s aims directly address limitations we identify in previous literature and they state what the paper achieves.**  *1. As the authors clarify that they are only interested in “estimating the prevalence of persistent undiagnosed, untreated, and uncontrolled hypertension”, the long presentation on the benefits of using longitudinal data in the introduction is unnecessary and misleading to the readers. I, therefore, suggest dropping the two paragraphs (second and third from last) in the introduction as that misleads the readers with very different expectations and is not consistent with the stated aim of the paper.*

**Response. In the first of the two paragraphs that are suggested to be dropped, we edited the text that identifies two of the limitations arising from analysis of cross-section data in order to align them with the stated aims of the study (see above). We dropped the third limitation previously identified in that paragraph (see above). In the second of the two paragraphs that is proposed to be dropped, we rewrote the first sentence to more accurately describe how the approach taken in this paper addresses the previously identified limitations in previous evidence (see above). We deleted the remainder of that paragraph. With these edits and deletions, the motivation is consistent with the stated aims of the study and what it delivers.**

*2. Please make it clear in the introduction that you are interested in “prevalence of persistent undiagnosed/untreated/uncontrolled hypertension”, not in “change in the prevalence of undiagnosed/untreated/uncontrolled hypertension”.*

***Response.* The introduction refers to persistent undiagnosed/untreated/uncontrolled hypertension and to hypertension that remains undiagnosed/untreated/uncontrolled (see quotations above). There is no mention of change in the prevalence of undiagnosed/untreated/uncontrolled hypertension.** *3. I suggest dropping or rephrasing the following sentence in the discussion section: “They do, however, provide sufficient cause to conduct similar analyses of longitudinal data in other countries to determine the extent to which undiagnosed, untreated, and uncontrolled hypertension are transient or persistent states and using this to identify policy priorities.” – since none of the limitations of cross-sectional analysis are substantially improved in this paper (as per the choice of parameters or interest), “similar analyses” of longitudinal data in other countries may not add much to the literature.*

**Response. We have rephrased this sentence.**

***“They may, however, motivate estimation of the prevalence of persistent undiagnosed, untreated, and uncontrolled hypertension in other countries.” – page 18***  *4. I suggest dropping or rephrasing the following sentence in the policy implications sub-section: “The longitudinal character of this study allows additional policy recommendations besides the implications one would derive from cross-sectional analyses.” Given the aim of this paper, very similar results (e.g., table 3) could have been produced using repeated cross-section data. I would suggest discussing the policy implications from the persistence (of undiagnosed/ untreated/ uncontrolled) perspective only.*

**Response. We have deleted this sentence and rewritten the one that followed it, as follows.**

***“Our estimates of substantial rates of persistent undiagnosed, untreated, and uncontrolled hypertension suggest that clinical and public health interventions are required to improve hypertension screening and care.” – page 23***

**We made a few other edits to the policy implications section of the discussion to improve alignment with the findings.**
